# Quantification of CDR1 Gene Expression in Fluconazole Resistant Candida Glabrata Strains Using Real-time PCR

**Published:** 2017-08

**Authors:** Shadi SHAHROKHI, Fatemeh NOORBAKHSH, Sassan REZAIE

**Affiliations:** 1. Dept. of Microbiology, Islamic Azad University, Varamin-Pishva Branch, Varamin, Iran; 2. Division of Molecular Biology, Dept. of Medical Parasitology and Mycology, School of Public Health, Tehran University of Medical Sciences, Tehran, Iran

**Keywords:** *Candida glabrata*, CDR1, Fluconazole resistance, Real-time PCR

## Abstract

**Background:**

The opportunistic fungi, particularly *Candida glabrata* has been known as main etiologic agents of life-threating infections in some patients. Although fluconazole is the most effective antifungal agent against candidiasis, *C. glabrata*, fluconazole-resistant strains have been increased recently overexpression or mutations of ATP-binding cassette (ABC) transporter family membrane proteins such as; Cg CDR1, Cg CDR2 are responsible for fluconazole resistance in a large proportion of candidiasis cases. The aim of this study was to evaluate CDR1 gene expression level as one of main mechanism involved in this resistance using.

**Methods:**

*Candida glabrata* strains were collected from various clinical samples in hospitals of Tehran in 2015 . After validation of all isolates by conventional and molecular methods, the susceptibility analysis to fluconazole of all isolates was performed using CLSI broth microdilution M27-A3 and M27-S4 protocols. Two isolates have been selected based on difference in susceptibility and CDR1-mRNA expression level of isolates was measured by Real-time PCR method.

**Results:**

Susceptibility results revealed that 32%, 64% and 4% of strains were susceptible, dose-dependent (DD) and resistant to fluconazole respectively. Furthermore, resistance strain of *C. glabrata* (MIC≥64 μg/ml) showed overexpression of CDR1 compared with sensitive strain in Real-time PCR analysis.

**Conclusion:**

Thus, it is necessary to investigate the functions of CgCDR1 genes as a transporter-related gene.

## Introduction

Since the early 1980s, fungi as a major human pathogen has been introduced, that induce serious disease especially among immunocompromised and hospitalized patients (1, 2). The incidence of invasive candidiasis has increased worldwide in recent decades (3–6).

Most of invasive fungal infections are associated with *Candida* species. Recently, increased prevalence of *C. glabrata* as a cause of serious infections has been reported from many hospitalized patients (6, 7). There is an increasing concern about rise in the number of *C. glabrata* in systemic infections, associated with high death rate (3, 8).

Several antifungal agents are available for treating candidiasis. Fluconazole, as a member of four major class of triazole, commonly used in clinical practice. The extensive use of fluconazole has resulted in development of resistant in *Candida* isolates. *C. glabrata* exhibits intrinsically low susceptibility to fluconazole and is often isolated from clinical specimen of AIDS or cancer patients (9, 10). Significant resistant to fluconazole was reported in clinical isolates of *C. glabrata*. Overexpression of ATP-binding cassette (ABC) transporter, was the prominent molecular mechanism involved in fluconazole resistance of *C. glabrata* isolates (9, 11, 12). In general, among ABC transporters, CgCDR1 and CgPDH1 play dominant roles in fluconazole resistance (13, 14). Azole resistance in *C. glabrata* was mainly associated with mutation or overexpression of CDR1 and CDR2 genes,” which encode transporters of the ATP-binding cassette (ABC) family, and the multidrug resistance (MDR1) gene, which codes for a major facilitator transporter” (15, 16). Recently, overexpression of CDR1 genes may be involved in the development of fluconazole resistance in mutant strains of *C. glabrata* (13, 17).

The aim of this study was to evaluate CDR1 gene expression level in Iranian strains of *C. glabrata* isolated from clinical samples as resistant by using Real-time PCR technique.

## Materials and Methods

Fifth-three *C. glabrata* strains have been recovered from various clinical samples in hospitals of Tehran in 2015 and initially, obtained isolates were identified by phenotypic methods such as colony on Candida Chromagar Medium (CHROMagar Company, Paris, France). In addition, we performed PCR-RFLP method as previously described (18). The verified *C. glabrata* strains were then cultured on Sabouraud dextrose agar (LAB M, Bury,UK) and incubated at 37 °C for 48 h.

The investigation of fluconazole susceptibility of *C. glabrata* strains has been performed according to clinical and laboratory standard institute (CLSI) document M27-A3 and M27-S4. Briefly, fluconazole was diluted in 4.2 g standard RPMI-1640 medium (Sigma Chemical Co), buffered to PH 7.0 with 17.265 g morpholinopropanesulfonic acid (MOPS) (Sigma) with L-glutamine without bicarbonate. The MICs endpoints of fluconazole as the level, induced a prominent reduction of growth (50% inhibition), compared to drug-free growth control. Based on this method, isolates with MICs≤8 μg/ml and MICs >64μg/ml were defined as fluconazole susceptible and resistant respectively, as well as isolates with MICs≤ 32 (μg/ml), were defined and selected as susceptible dose-depended (19). One sensitive strain as control as well one resistance strain were chose for performing our complementary tests. *C. parapsilosis* (ATCC22019) was run as a quality control in parallel with every run of MICs plates.

RNA molecules were extracted from cells during the log phase of growth at 48 h of incubation by using Ferments kit according to the manufacturer′ s instruction (Ferments, Vilnius, Lithuania). DNA contamination within the samples is removed during the mRNA isolation using DNase I.

The quality of RNA was checked using a spectrophotometer by which the ratio of A269 nm: A280 nm was shown as 1.8–2.0, which corresponded to 90%–100% pure nucleic acid.

CDNA molecules were synthesized using cDNA reverse transcription kit following the manufacturer′ s instructions (Vivantis Company, Malaysia).

The 21 nt primers were designed using primer design BLAST software (CDR1-S1: 5′- Ggtgctaatatccaatgttgg -3′, CDR1-aS1: 5′- Gtaatggttctctttcagctg -3′) and were synthesized by Sinagene (Tehran, Iran) for analysis of the *C. glabrata* CDR1 gene expression.

The expression of CDR1 was examined by quantitative real-time PCR. Amplification was performed in 8-tube strip contained 10 μl 1×SYBER Green PCR Master mix, 0.8 pmol ml-1 of each forward and reverse primer and 2 μl template cDNA in a final volume of 20 μl. Tests were performed in triplicate.

Cycling conditions included an initial step at 94 °C for 10 min, followed by 40 cycles of 94 °C for 15 sec, 55 °C for 20 min and 72 °C for 20 sec. The beta-actin gene was used as a normalizer and expression level of CDR1 gene was calculated by the 2-ΔΔCt method.

## Results

Most of isolates recovered from Sputum samples (45%). In addition, 24.4% and 22.6% of the isolates were from bronchoalveolar lavage (BAL) and vagina samples respectively. The results of MIC for fluconazole in all investigated isolates were presented in Table 1. Out of 53 tested isolates, 17 (32%), 34 (64%) and 2 (4%) strains could be considered as susceptible (MIC≤ 8μg/ml), susceptible dose dependent (MIC= 16–32 μg/ml), and resistant (MIC≥ 64μg/ml) respectively (Table 2). The MIC_50_ and MIC_90_ of the isolates have also been indicated in same table.

**Table 1:** Antifungal susceptibility pattern of *C. glabrata* strains

**Species**	**Antifungal**	**MIC_50_ (μg/ml)**	**MIC_90_ (μg/ml)**	**Range (μg/ml)**	**Susceptible**	**Dose Dependent**	**Resistance**
*Candida glabrata*	Fluconazole	16	32	0.5–64	32% (17)	64% (34)	4% (2)

**Table 2:** The results indicate the significant induction of mRNA expression level in fluconazole- resistant strain (CDR1r) compared to control strain (CDR1c)

**Gene**	**Type**	**Reaction Efficiency**	**Expression**	**Std. Error**	**95% C.I.**	**P(H1)**	**Result**
b actin	REF	1.0	1.000	1.000 – 1.000	1.000 – 1.000	0.000	---
CDR1c	TRG	1.0	1.000	1.000 – 1.000	1.000 – 1.000	0.000	---
CDR1r	TRG	1.0	64.000	64.000 – 64.000	64.000 – 64.000	0.000	UP

Legend: TRG – Target, REF – Reference

**Fig. 1:**
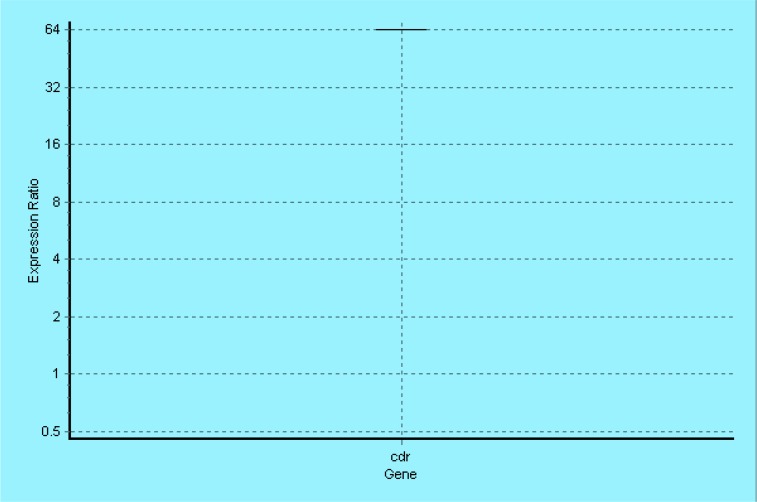
The real-time RT-PCR assay of *CDR1* mRNA expression which indicates overexpression of *CDR1* mRNA

Furthermore, CDR1 mRNA levels were measured in order to investigation the mechanism of fluconazole resistance in both sensitive as well as resistant isolates of *C. glabrata* by quantitative real-time RT-PCR assay. Based on the obtained data, relative quantification of CDR1 mRNA level was calculated to be 64.00 after normalization to a housekeeping gene (beta-actin). The mention results indicate the significant induction of mRNA expression level in fluconazole-resistant strain compared to susceptible strain as control (Fig. 1).

## Discussion

Candida species is one of the primary fungal pathogen causes of human infection. Recently, *C. glabrata* has been emerged as the main cause of life-threatening infections. The major mechanisms of fluconazole resistance among *C. glabrata* species described to be based on CgCDR1, CgCDR2, as well as ergosterol synthesis pathway enzymes (20).

In this study, we developed a real-time PCR method for quantification CDR1 gene expression as one of the ABC transporter family membrane genes, which play a pivotal role in fluconazole resistance in both fluconazole-sensitive and fluconazole resistant strains of *C. glabrata*. Obtained results indicate higher expression in resistance strain compared to the susceptible one.

Plasma membrane proteins Cdr1p and Cdr2p are an important factor in fluconazole resistance in *Candida*, encoded by ABC transporter genes, CDR1 and CDR2 (21).

The results of this study same as Yoo et al. revealed that the main reason for fluconazole resistance in *C. glabrata* may be related to CgCDR1 gene. Many genes of *C. glabrata* have been duplicated in various regions of the genome (22). Duplication accomplish was suggested in chromosomal sections that encode some ABC transporter genes, CgCDR (22).

Similarly, recombinations in tandem repeat genes, which encode cell wall proteins, explain probably an adaptive function, which causes a macroevolution in asexual species of fungi (23).

Fluconazole resistance in *C. glabrata* strains induced by overexpression of transporter family genes has an important role in transport of fluconazole (22).

The results of this study are according to the data observed in the investigations, performed on fluconazole resistant *C. glabrata* strains (21, 24, 25).

## Conclusion

We identified the CDR1 gene regulation in fluconazole-resistant strains. However, the exact mechanism of resistance mediation has not been answered. Thus, it is necessary to investigate the functions of CgCDR1 genes as a transporter-related gene.

## Ethical considerations

Ethical issues (Including plagiarism, informed consent, misconduct, data fabrication and/or falsification, double publication and/or submission, redundancy, etc.) have been completely observed by the authors.
